# Congenital Intranasal Glioma

**DOI:** 10.1155/2011/175209

**Published:** 2011-08-23

**Authors:** Sajad Ahmad Salati, Ajaz Ahmad Rather

**Affiliations:** ^1^Department of Plastic and Reconstructive Surgery, King Fahad Medical City, Riyadh 11525, Saudi Arabia; ^2^Department of Surgical Specialties, Sheri Kashmir Institute of Medical Sciences, Soura, Srinagar 190020, India; ^3^Department of General Surgery, Sheri Kashmir Institute of Medical Sciences (Medical College), Bemina, Srinagar 190018, India

## Abstract

Congenital midline swellings of nose are encountered rarely, and nasal gliomas constitute about 5% of such lesions. Various theories have been suggested to explain the pathogenesis. Imaging preferably by MRI is mandated to study the extent and to rule out intracranial extension. Treatment is complete excision, and the approach depends upon the extent of the lesion and availability of expertise. We present the management of one such case of congenital intranasal glioma without any intracranial extension that presented as a septal polyp.

## 1. Case Presentation

**Figure 1:**
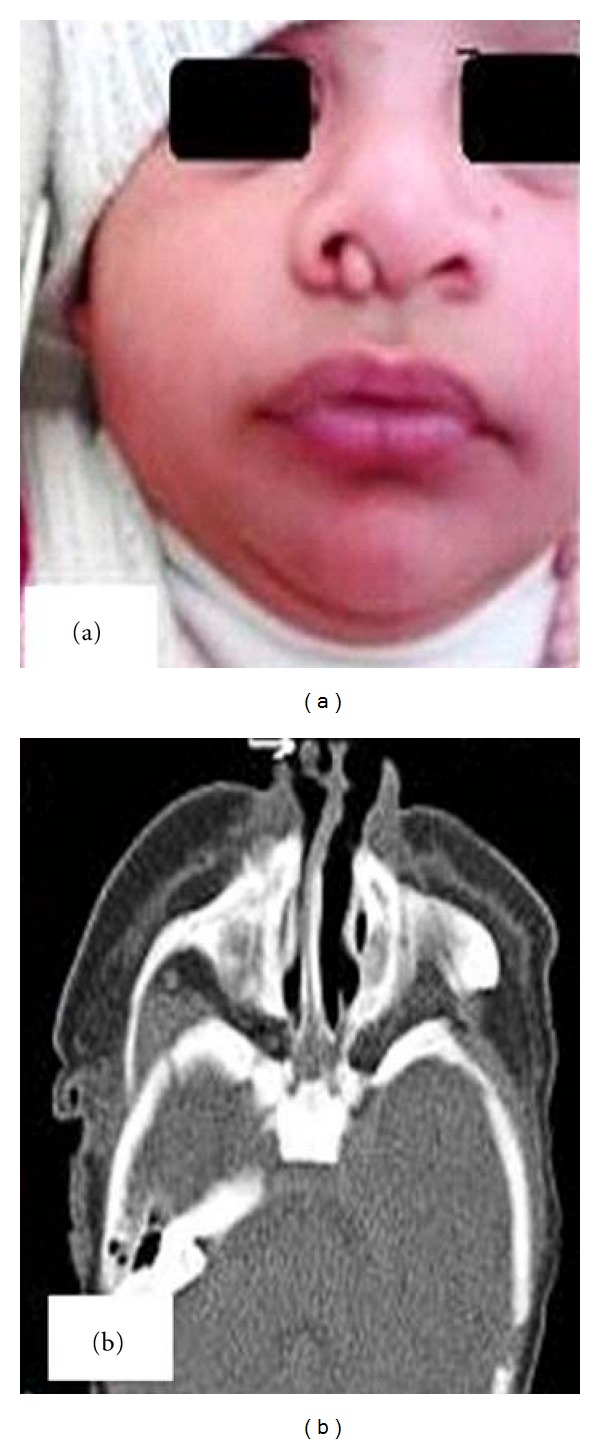
(a) 3 months old baby with right nasal congenital polyp-glioma. (b) Axial CT scan revealing a soft tissue lesion arising from nasal septum (white arrow).

 A three-month-old male baby was brought by parents with complaint of a fleshy mass coming out of right nostril ever since birth. There was history of occasional nasal obstruction, and the mass was growing slowly. There was no history of epistaxis or any other nasal discharge. The baby was first in birth order, product of nonconsanguineous marriage and born at fullterm by a normal vaginal delivery. The family history was unremarkable. On general physical examination, the baby was playful and hemodynamically stable. Examination of nose revealed a lesion protruding through the right external nares like a polyp (Figure 1(a)). The polyp was soft, pedunculated, nonpulsatile, and moving in and out with respiration, and the skin over it had no special features. The pedicle was arising from caudal part of nasal septum. No change in size of the mass was observed during crying or on jugular vein compression (Furstenberg's test). The left nostril was patent. There were no other abnormalities. The parents were counseled, and CT scan was advised to study the nature and extent of the polyp. CT scan revealed a well-defined soft tissue attenuation lesion, about 10 mm × 8 mm in dimensions, at the caudal end of nasal septum in right side with a stalk (Figure 1(b)). No bony defect or intracranial extension or other synchronous lesion was seen. As the base of the pedicle was clearly visible, trans-nasal excision was done under general anesthesia. There were no perioperative complications, and the patient was particularly observed for postoperative bleeding, CSF leak, fever or other features of infection. Histopathological analysis of the specimen revealed nonmalignant gliomatous cells with low proliferative activity. No meningeal or dural tissue was identified. The diagnosis of nasal glioma was hence established. The patient remained under followup for fifteen months and did not show any evidence of recurrence of the lesion.

## 2. Discussion

Congenital midline nasal masses are rare anomalies that occur in about one in 20000–40000 live births [1]. Nasal gliomas account for approximately 5% of all congenital nasal swellings. These usually arise during infancy or later childhood with relative peaks of occurrence between 5 and 10 years of age. Although the majority of patients present during the first year of life, a later presentation may be due to a specialist's failure to recognize a subclinical lesion in childhood. 

The term “nasal glioma” is a confusing misnomer as it implies a neoplastic condition, which it is not. It needs to be differentiated from glioma, which is a malignant tumor of the brain [1, 2]. 60% of these gliomas are extranasal, lying external to the nasal bones and cavities; 30% are intranasal lying within the nasal cavity, mouth, or pterygopalatine fossa and 10% are mixed, dumbbell shaped communicating through a defect of the nasal bones [3]. Our case was of an intranasal glioma. 

The possible theories of development of nasal gliomas include the following: (a) sequestration of glial tissue of the olfactory bulb entrapped during cribriform plate fusion; (b) ectopic neural tissue cells; (c) encephaloceles with lost intracranial connection and meningeal continuity; (d) inappropriate closure of the anterior neuropore (fonticulus frontalis) [1, 4]. In 15–20% of cases, a fibrous stalk exists to connect them to the intracranial space.

Clinically, these masses are firm in consistency, noncompressible, nonpulsatile, grayish or purple lesions. These masses can protrude through the nostrils and can be confused with a nasal polyp. The patient may suffer from nasal obstruction, epistaxis, cerebrospinal fluid (CSF) rhinorrhoea, nasolacrimal duct obstruction, epiphora, hypertelorism and cosmetic deformity [5].

Histologically, these lesions are made up of astrocytic neuroglial cells interlaced with fibrous and vascular connective tissue [5] that may be covered with skin or nasal respiratory mucosa. True capsule is absent, and mitosis is rare. The glial nature of the cells can be further confirmed by immune-histochemical demonstration of S100 protein and GFAP.

CT scan or MRI forms the mainstay of investigation as fine needle aspiration cytology or excision biopsy carries a significant risk of meningitis or CSF leaks [6]. CT scan demonstrates bony defects, and MRI can demonstrate the characteristics of the soft-tissue mass and its possible intracranial connection. On CT, the mass is usually isodense to brain tissue. On MRI, the lesion is isointense to hypointense relative to gray matter on T1-weighted sequences and hyper-intense on T2-weighted and proton density sequences [5, 7]. Magnetic resonance imaging also has an advantage of minimizing the level of exposure to ionising radiation, particularly in infants.

The clinical differential diagnosis of nasal gliomas includes several disorders, which can present as nasal masses [1, 8]. Some of such important lesions include: (a) nasal dermoids, which constitute the most common congenital nasal anomaly and are cavities or sinus tracts possessing epithelial lining and variable numbers of skin appendages, including hair follicles, sebaceous glands, and eccrine glands; (b) encephaloceles which constitute the lesions caused by herniation of neural tissue through defects in the skull. They may contain meninges (meningocele) or brain matter and meninges (encephalomeningocele), or they may communicate with a ventricle (encephalomeningocystocele). Encephaloceles are etiologically similar to nasal gliomas as per one of the theories; (c) hemangioma which are the most frequent benign vascular tumors in infancy. 

 The treatment of choice of nasal gliomas is complete surgical excision [1]. Gliomas are benign but incomplete excision results in a 4% to 10% recurrence rate. The approach depends upon the location and extent of the lesion [1] and levels of expertise available. When facilities are available, intranasal endoscopic surgery is considered most appropriate approach for the removal of intranasal glioma having no intracranial extension as this approach allows precise excision with minimal trauma to the surrounding tissues [9]. If however intracranial extension is evident, than frontal craniotomy, multidisciplinary team approach may be required [10] in specialized neurosurgical or craniofacial centers to ensure complete and safe excision of the lesions.

## 3. Conclusion

Nasal gliomas are rare congenital anomalies. It is mandatory to rule out intracranial extension by cross-sectional imaging, preferably by MRI before performing any invasive procedure. Surgical excision in the mainstay of treatment and the route depend upon the extent of the lesion and the available expertise.

##  Conflict of Interests

The authors declare that there is no conflict of interests.
